# SMAdd-seq: probing chromatin accessibility with small molecule DNA intercalation and nanopore sequencing

**DOI:** 10.1093/nar/gkaf671

**Published:** 2025-07-19

**Authors:** Gali Bai, Namrita Dhillon, Colette Felton, Brett Meissner, Brandon Saint-John, Robert Shelansky, Elliot Meyerson, Eva Hrabeta-Robinson, Babak Hodjat, Hinrich Boeger, Angela N Brooks

**Affiliations:** Department of Biomolecular Engineering, University of California, Santa Cruz, Santa Cruz, CA 95064, United States; Department of Biomolecular Engineering, University of California, Santa Cruz, Santa Cruz, CA 95064, United States; Department of Biomolecular Engineering, University of California, Santa Cruz, Santa Cruz, CA 95064, United States; Department of Molecular, Cell, and Developmental Biology, University of California, Santa Cruz, Santa Cruz, CA 95064, United States; Department of Biomolecular Engineering, University of California, Santa Cruz, Santa Cruz, CA 95064, United States; Department of Biomolecular Engineering, University of California, Santa Cruz, Santa Cruz, CA 95064, United States; Department of Molecular, Cell, and Developmental Biology, University of California, Santa Cruz, Santa Cruz, CA 95064, United States; Cognizant AI Lab, San Francisco, CA 94105, United States; Department of Biomolecular Engineering, University of California, Santa Cruz, Santa Cruz, CA 95064, United States; Cognizant AI Lab, San Francisco, CA 94105, United States; Department of Molecular, Cell, and Developmental Biology, University of California, Santa Cruz, Santa Cruz, CA 95064, United States; Department of Biomolecular Engineering, University of California, Santa Cruz, Santa Cruz, CA 95064, United States

## Abstract

Studies of *in vivo* chromatin organization have relied on the accessibility of the underlying DNA to nucleases or methyltransferases, which is limited by their requirement for purified nuclei and enzymatic treatment. Here, we introduce a nanopore-based sequencing technique called small-molecule adduct sequencing (SMAdd-seq), where we profile chromatin accessibility by treating nuclei or intact cells with a small molecule, angelicin. Angelicin preferentially forms photoadducts with thymine bases in linker DNA, thereby labeling accessible DNA regions. By applying SMAdd-seq in *Saccharomyces cerevisiae*, we demonstrate that angelicin-modified DNA can be detected by its distinct nanopore current signals. To systematically identify angelicin modifications and analyze chromatin structure, we developed a neural network model, NEural network for mapping MOdifications in nanopore long-reads (NEMO). NEMO accurately called expected nucleosome occupancy patterns near transcription start sites at both bulk and single-molecule levels. We observe heterogeneity in chromatin structure and identify clusters of single-molecule reads with varying configurations at specific yeast loci. Furthermore, SMAdd-seq performs equivalently on purified yeast nuclei and intact cells, indicating the promise of this method for *in vivo* chromatin labeling on long single molecules to measure native chromatin dynamics and heterogeneity.

## Graphical Abstract

**Graphical Abstract:**
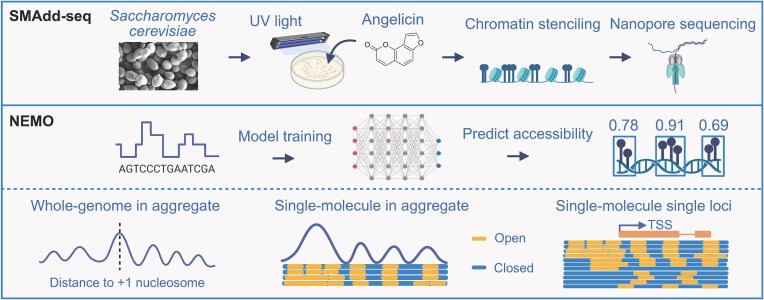

## Introduction

DNA in all eukaryotic cells is packaged into nucleosomes. This nucleoprotein complex together with DNA-binding proteins and RNA comprises chromatin. The dynamic and variable nature of chromatin regulates all DNA-centric processes and plays a vital role in cell growth, differentiation, and development. Nucleosomes are composed of ∼147 bp (∼1.7 turns) of DNA wrapped around a central histone protein octamer. Arrays of nucleosomes separated by ∼20–90 bp of linker DNA appear as beads on a string in electron micrographs [1]. Nucleosomes block access of DNA binding factors to the underlying DNA and impede transcription, replication, DNA repair, and recombination machineries [2]. The distribution of nucleosomes across the genome is not uniform and varies significantly between open and closed chromatin. There is also considerable heterogeneity in nucleosome distribution at different gene loci in open chromatin and also within each gene [3]. This chromatin structure varies with growth conditions, differentiation, and development [4]. Thus, knowledge of the dynamic chromatin landscape can yield important insights into development, disease, and drug response.

Assays to determine nucleosome distribution at specific gene loci were developed soon after the discovery of the nucleosome [5]. The original assays probed for accessibility of chromatin to DNA endonucleases that mostly cleave linker DNA [6, 7]. These were subsequently adapted to genome-wide nucleosome distribution studies using short-read Illumina sequencing leading to MNase-seq [8], DNase-seq [9], and ATAC-seq [10], among others. While nucleosome distribution profiles from short-read data have been vital to our understanding of chromatin structure and function, they only provide an aggregate view of nucleosome distribution across all cells in the population. A granular view of the heterogeneity in nucleosome spacing in individual cells is lacking in these short-read data. Also absent is a view of coordination of nucleosome organization across long genomic distances. Short-read data also suffer from biases introduced by PCR amplification, read mapping, and DNA fragmentation [11].

A more recent advancement in sequencing was the development of long-read nanopore sequencing technology, where an electrical current is passed across a biological pore embedded in a lipid bilayer. As single-stranded DNA is channeled through the pore by a motor protein, the electrical current undergoes shifts based on the sequence of the six bases of DNA (*k*-mer) present in the pore at any given time [12]. Modified DNA bases can also be detected from electrical shifts from nanopore sequencing [13] leading to the development of single-molecule long-read assays to map chromatin accessibility using DNA methyltransferases (MTase). Long-read sequencing approaches allow for the detection of modified DNA without the bias of PCR amplification and can also detect endogenous and exogenous DNA modifications such as 6mA and 5mC. Data from these methods have yielded novel insights into genome organization, single-molecule nucleosome distribution in the genome, cell-to-cell heterogeneity, and gene regulation [14–20]. One drawback of MTase assays in mapping nucleosome and transcription factor occupancy with long-read sequencing is that they require extraction of nuclei from cells for MTase treatment, rendering the process arduous and also subject to a disrupted chromatin state [21, 22].

To develop a method that allows for accurate, *in vivo* chromatin labeling, we explored the use of small molecules to map accessible chromatin. Small molecules have long been used to investigate chromatin structure [23]. One such molecule, the furocoumarin, psoralen intercalates between double-stranded nucleic acids and undergoes photocycloaddition with thymine/uracil pyrimidine bases when exposed to UVA light to form DNA/RNA adducts [24, 25]. Psoralen adducts preferentially occur in linker DNA in chromatin and this property has been widely applied in structural DNA and chromatin accessibility studies [24]. For instance, we previously used psoralen cross-linking of linker DNA in electron microscopy (EM) studies to highlight the stochastic positioning of nucleosomes on *PHO5* promoter molecules [3]. Due to its extended structure, psoralen forms both covalent mono- and di-adducts with one or both strands of a nucleic acid helix, with the latter forming interstrand cross-links on double-stranded molecules. The psoralen family of molecules exhibits a 5′-TA > 5′-AT > 5′-TG > 5′-GT dinucleotide preference for DNA cross-links [26], with 5′-TA dinucleotides being significantly preferred.

Angelicin is an isomer of psoralen with an atomic mass of 186 g/mol (similar to glucose). Like psoralen, angelicin has been shown to preferentially form adducts with DNA that is not bound by nucleosomes or transcription factors [25]. Unlike psoralen, angelicin, due to its angular structure, has been described to form photo-monoadducts with one of the two DNA strands [25, 27]. While some studies report that angelicin can slowly form covalent interstrand cross-links upon prolonged UV irradiation (1 h), the authors also recommend its application for photoinduced labeling of chromatin in instances where covalently cross-linked DNA strands are problematic [28]. Furthermore, angelicin easily traverses cell membranes and thus can be applied to the analysis of chromatin structure with little perturbation of the cells [24–25, 29]. We exploited the cell permeability of angelicin as well as its ability to primarily form photoadducts on single strands of accessible DNA to assess whether we can detect these adducts in open chromatin using nanopore sequencing.

Here we report the development of small-molecule adduct sequencing (SMAdd-seq) that utilizes angelicin to map chromatin accessibility with nanopore sequencing. We show that intercalation of angelicin causes a detectable shift in the nanopore current signal and have developed a neural network approach to predict angelicin modification from these signal data. We can accurately detect known chromatin accessibility patterns in angelicin-modified nuclei and intact cells (spheroplasts) in *Saccharomyces cerevisiae* and can discern single-molecule chromatin profiles and regulatory patterns at individual loci.

## Materials and methods

### Yeast strains and culture

The *S. cerevisiae* strain YS18 (MATalpha his3-11 his3-15 leu2-3 leu2-112 can1-100 ura3Δ5) (S288C derivative) was used in this study. Cells were grown in YPD (1% yeast extract, 2% peptone, 2% dextrose) at 30°C.

### Yeast spheroplast preparation and nuclei isolation

Yeast spheroplast preparation and nuclei isolation were carried out as described previously [30].

### Angelicin modification of yeast and genomic DNA extraction

Yeast chromatin was modified with angelicin using either purified nuclei or yeast spheroplasts. Spheroplasts were prepared from 250 ml of logarithmically growing cells (∼1.5 × 10^7^ cells/ml) cultured in YPD. Prepared spheroplasts were washed twice in TMSorbitol buffer (1.2 M sorbitol, 20 mM Tris–HCl, pH 8.0, 1 mM MgCl_2_) and resuspended in 500 μl TMSorbitol buffer. Fifty microliters of a 10 mM angelicin (Sigma, A0956, prepared in 100% ethanol) stock solution was diluted into 490 μl of TMSorbitol buffer and added to the spheroplast suspension for a final angelicin concentration of 500 μM. Following a 5-min incubation at room temperature, 250 μl of the suspension was distributed into 3 wells of a 12-well plate (∼1 × 10^9^ spheroplasts/well) and nestled on a surface of ice/water. The spheroplast_angelicin suspension was subjected to seven rounds of a 5-min exposure to 365 nm UVA light in a UV Stratalinker (Stratagene UV Stratalinker 2400, power 5.0 fitted with 4 EIKO F15T8/BL bulbs) alternating with a 5-min incubation (in the dark) on ice/water to form covalent thymine–angelicin bonds.

Nuclei were prepared as previously described [30] from logarithmically growing yeast cells (∼1.5 × 10^7^ cells/ml) cultured in YPD. Two aliquots of nuclei (each containing ∼5 × 10^8^ nuclei) were pooled, spun, and resuspended in 400 μl TM buffer (20 mM Tris–HCl, pH 8.0, 1 mM MgCl_2_). Forty microliters of a 10 mM angelicin stock (in 100% ethanol) was diluted in 360 μl of TM buffer and added to the nuclei suspension for a final angelicin concentration of 500 μM. Following a 5-min incubation at room temperature, 250 μl of the nuclei–angelicin mix was distributed into 3 wells of a 12-well plate (∼3 × 10^8^ nuclei/well) and subjected to UV light as above.

Following UV irradiation, the contents of all wells were pooled into a 1.5-ml DNA low-adhesion tube, the wells were washed with 100 μl of ice-cold TMSorbitol (spheroplasts) or TM (nuclei) buffer, and added to the same low-adhesion tube to maximize retrieval. High molecular weight DNA was purified using the NEB Monarch HMW DNA Extraction Kit for Tissue (T3060L). Wide-bore pipette tips were used to minimize shearing of modified and unmodified high molecular weight DNA. The purified DNA was quantified using the DNA Broad Range Kit for Qubit (Thermo Fisher) and also analyzed on a genomic DNA ScreenTape on a TapeStation 4150 (Agilent Technologies). Positive and negative control data for the neural network training were generated from purified high molecular weight DNA (∼6 μg) that was incubated with or without (mock treated with ethanol) 500 μM angelicin, respectively, followed by UV treatment as described above.

### Oxford nanopore sequencing

Three to four micrograms of high molecular weight DNA was used to prepare genomic libraries for sequencing with Oxford Nanopore Technologies (ONT) SQK-LSK110 kits for use with R9.4.1 (FLO-MIN106) flow cells. Approximately 1.5 μg of the library was loaded onto flow cells, and all library sequencing was undertaken on a MinION for 24 h each with MUX scanning every 6 h to extend the life of the flow cell.

### Basecalling and aligning sequencing data

The data were basecalled and aligned to sacCer3 genome with Dorado v0.6.2 using pore model dna_r9.4.1_e8_sup@v3.6 and parameters --emit-moves [31, 32]. Reads were aligned, sorted, and indexed using Samtools v1.13. Secondary and supplemental reads were further filtered. Uncalled4 v4.1.0 was run to align signals to *k*-mers and obtain eventalign output [33, 34].

### Alkaline agarose gel electrophoresis

Agarose gel electrophoresis was performed on plasmid pBlueScript DNA modified with 0, 100, 200, 500, or 1000 μM angelicin and digested with Not1 according to [35]. The modified DNA was purified on SPRI beads and resolved on a 1% alkaline agarose gel. DNA was visualized and documented on a Bio-Rad ChemiDoc XRS imager after ethidium bromide staining and destaining.

### Identification of *k*-mer signal distribution peaks and informative *k*-mers

We aggregated the mean signal value for each *k*-mer (6-mer) in each read from the eventalign file. For each *k*-mer, the signal density was calculated using numpy np.histogram function at the range of 25–150. We then used scipy.signal.find_peaks with parameters prominence = 0.005 and distance = 5 to identify *k*-mers with a secondary peak in the positive control sample. We considered these *k*-mers informative *k*-mers to indicate signal shifts produced by modification. We also identified peaks in the negative control sample and found that no *k*-mers had more than one peak in that sample. We then generate sequence logos using all 169 informative *k*-mers [36].

### Picoamp signal preprocessing for model training

Signal picoamps with a value smaller than −50 or larger than 150 were clipped to −50 and 150. Signals were normalized by the mean and standard deviation within the data input to the model. In each read from each sample, picoamp signals were scanned by a sliding window of 400 with a step size of 1. Input signals of length 400 are represented as a one-dimensional array [1, 2, 3, 4, 5, …, 400]. For every data point, a single signal shift was applied to capture the sequential nature of nanopore signals (e.g. [2, 3, 4, 5, 6, …, 401]).

### Training, validation, and testing of the neural network model

We developed NEMO (a NEural network model for mapping MOdifications in nanopore long reads), a computational tool for training and predicting angelicin modification sites. NEMO is implemented in PyTorch (v2.0.1) [37] and utilizes a Residual Network classifier optimized for one-dimensional signal data analysis [38, 39]. Each positive and negative control dataset was divided into train (60%), validation (20%), and test (20%) sets. Positive control data were labeled with prediction probabilities of 1.0, and negative control data were labeled with prediction probabilities of 0.0. The model was trained over 100 epochs, with a batch size of 512 and 1000 batches per epoch. For training model parameters with gradient descent, binary cross-entropy loss was calculated using the function torch.nn.BCELoss after each step, and model parameters were updated with the function torch.optim.Adam [40]. Following each epoch of training, model performance was evaluated in the validation set with batch size of 512 and 500 batches per epoch. Training accuracy, training loss, validation accuracy, and validation loss were recorded per epoch. The model with the highest validation accuracy after 100 epochs was saved as the optimal model for further analyses.

This final model was applied to classify signals in the test dataset, where standard performance metrics, including true positive rate (TPF), false positive rate (FPR), true negative rate, false negative rate, accuracy, precision, recall, and *F*1 score, were calculated to comprehensively assess the model’s performance. Receiver operating characteristic (ROC) and AUC scores were generated using the roc_curve and auc functions in scikit-learn [41].

### Neural network prediction in chromatin sequencing data

The model trained on control datasets was applied to predict angelicin modifications in normalized chromatin sequencing data. First, for each signal sequence mapped to a single read, NEMO scans the signals with a sliding window of 400, moving in steps of 200. Then, each length-400 signal was fed into the neural network model to receive a modification probability score, which is assigned to all genomic positions within that window based on Dorado basecaller’s sequence-to-signal mapping (∼30–50-bp regions). Finally, read modification scores assigned to the same genomic position are averaged to produce the final modification score for that position.

### Aggregate analysis of +1 nucleosomes dyad

For aggregate analyses, we used 5440 annotated +1 nucleosome dyads across the yeast genome [42]. For each gene, we calculated the average modification probability for positions up to 2000 bp upstream and downstream relative to each +1 nucleosome. Finally, the scores from an individual gene locus were averaged to generate genome-wide aggregated modification scores at +1 nucleosome dyads. PacBio methylation data were downloaded from GSE243114 [43]. The bam files were parsed by pysam [44–46] to obtain base-level modifications, which were further averaged for positions up to 2000 bp upstream and downstream relative to each +1 nucleosome. For visualization, all averaged scores were further normalized by mean and standard deviation.

### Quality control of chromatin sequencing data

We used the observance of expected nucleosome periodicity as a quality control (QC) metric for the ability of the trained model to predict chromatin accessibility in each chromatin-modified sample. From three replicates of angelicin-treated nuclei, nucleosome occupancy patterns in replicate 3 showed a poor Pearson correlation with the expected nucleosome periodicity and failed to show the expected nucleosome patterns in aggregates, thus did not pass our QC (Supplementary Table S2).

### Identification of genes with well-positioned nucleosomes at transcription start sites

To identify genes with well-positioned nucleosomes at their promoters, we calculated the Pearson correlation coefficient between each gene’s individual angelicin modification scores and the genome-wide aggregated scores within 600 bp upstream and downstream of the +1 nucleosome dyads. In total, we examined 5440 genes that have annotated +1 nucleosomes dyads [42]. We also characterized the heterogeneity of nucleosome positioning at gene transcription start sites (TSS) by calculating the variance of angelicin modification scores between reads for each gene (Supplementary Table S2). Genes were first ranked by the highest Pearson correlation to the lowest, then by the height read coverage to the lowest, and by least heterogeneity to the largest. We used a 75% percentile cutoff for each sample to select genes with a high Pearson correlation, high coverage, and low variability. We identified 380 genes in replicate 1, 230 genes in replicate 2, 59 genes in replicate 3, and 57 genes in spheroplast data. Because of the low overall sequencing coverage and high variability among genes with greater coverage, only 38 high-coverage genes were consistently detected in the first two replicates and were therefore used for visualization.

### Single-molecule clustering and visualization

In NEMO, we implemented a genome track visualizer using matplotlib v3.6.2 [47] to show modifications in individual reads. Reads covering a minimum of 80% of given genome regions were used to construct a modification probability matrix. Highly modified reads were identified as reads with an average modification score of 0.8 and were filtered out. Missing values in the matrix were imputed with scikit-learn v1.1.2 simpleImputer function under “most_frequent” strategy. The matrix was then input to the scikit-learn *k*-means clustering algorithm, where reads are clustered based on their modification profiles. We initially set *k* = 2 for clustering reads per gene and increased *k* if visual inspection revealed distinct subclusters. Clustering was performed with random centroid initializations, and the cluster ids are collected after 300 iterations. Single molecules were colored based on their predicted angelicin modification scores ranging between a probability of 0 and 1, with dark blue indicating 0% probability of angelicin modifications, thus nucleosome occupied regions, while bright yellow indicating a 100% probability of angelicin modifications and thus accessible regions.

To visualize 38 genes with well-positioned nucleosomes at TSSs, reads mapped to each gene promoter region (1200 bp centered at +1 nucleosome dyads) were stacked together. Genes were ordered from highest to lowest Pearson correlation coefficient. Reads within each gene were ordered by *k*-means clustering. Aggregated angelicin modifications were calculated by averaging scores across reads for each gene, and then averaging across the genes. The averaged scores were further normalized by minimum–maximum value.

To visualize individual gene locus, reads mapped to *zz-YIL161W* (chrIX:38868–40068), *FEX2* (chrXVI:13765–14965), *CLN2* (chrXVI:66400–67550), and *NUP170* (chrII:74300–75800) are clustered and visualized using NEMO with the method described above. Aggregated angelicin modifications for each cluster were calculated by averaging scores across the reads for each cluster and plotted on top of each cluster. The averaged scores were further normalized by minimum–maximum value.

## Results

### Angelicin modification and sequencing of DNA

**Figure 1.**
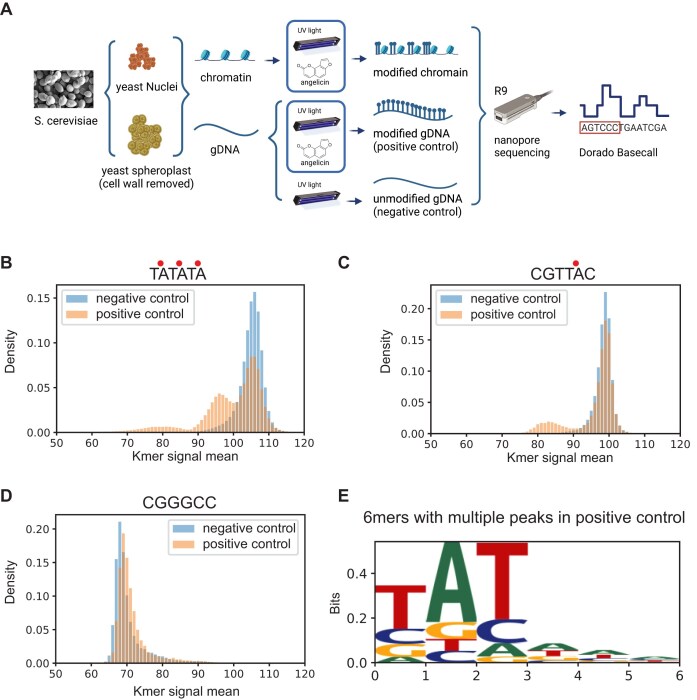
SMAdd-seq: a method using angelicin modification to probe chromatin accessibility. (**A**) Schematic of the SMAdd-seq method. Yeast nuclei and spheroplasts were treated with 500 μM angelicin and then exposed to multiple rounds of UV light to cross-link the angelicin with DNA. The modified DNA was extracted and sequenced by nanopore sequencing with R9 flow cells, and then basecalled with Dorado. Yeast genomic DNA was isolated and treated with 500 μM angelicin under UV light or UV light only to produce positive and negative control samples. Created in BioRender. Bai, G. (2025) https://BioRender.com/s77q637 (B–D) Histograms of the nanopore signal currents produced from a given *k*-mer in yeast DNA that had been treated either with UV light only (blue: negative control) or with angelicin and UV (orange: positive control). Dots indicate the inferred angelicin cross-linking sites. (**B**) A modifiable *k*-mer TATATA has two shifted peaks in the positive control sample, (**C**) a modifiable *k*-mer CGTTAC has one shifted peak in the positive control sample, and (**D**) an unmodifiable *k*-mer CGGGCC has no shifted peak in the positive control sample. (**E**) Sequence logo for the 169 *k*-mers with shifted peaks in the positive control sample.

To determine whether thymine bases modified by angelicin could be detected by nanopore sequencing, we first tested naked DNA that had been treated with angelicin and UV. High molecular weight yeast genomic DNA was treated with 0, 20, 100, or 500 μM angelicin and exposed to UV-A at 365 nm for photocycloaddition (Fig. 1A, Supplementary Fig. S1A, and Supplementary Table S1). To ensure high levels of angelicin modification, we used the same parameters as those previously optimized [3] for mapping nucleosomes *in vitro* with psoralen cross-linking (Supplementary Fig. S1B). These samples were then used to prepare libraries for nanopore sequencing using the DNA ligation kit SQK-LSK110 (Oxford Nanopore Technologies) and were sequenced on R9.4.1 Minion flow cells. All reads were basecalled and mapped to the yeast SacCer3.0 genome with Dorado 0.6.2 [48] and then aligned to the yeast SacCer3.0 genome with Uncalled4 eventalign [34] (Fig. 1A).

While we were able to sequence angelicin-modified DNA through the nanopore, we noticed that the sequencing output of angelicin-modified DNA was lower than unmodified DNA and the pores became inactive considerably faster (Supplementary Fig. S2A–C). Given that our preliminary structural analysis showed thymine bases modified by angelicin could easily fit through a nanopore (data not shown), as well as the fact that we were able to sequence some angelicin-modified DNA, it was unlikely that the angelicin monoadduct itself was blocking the pores. Although previous work has shown that angelicin should form covalent bonds with a single thymine base on one DNA strand, without forming cross-links between the two strands of DNA [25, 27], a study from Lown and Sim showed that a small population of cross-linked molecules is also generated by angelicin, albeit in a much lower proportion compared to psoralen [28]. To assess whether angelicin induced DNA cross-links in our treatment protocol, we asked whether we could visualize cross-links in angelicin-modified plasmid DNA on a denaturing alkaline agarose gel. This technique denatures DNA into single strands and DNA molecules with cross-links are unable to separate into single strands and thus run slower than their single-stranded counterparts. (Supplementary Fig. S2D). We treated plasmid DNA with varying concentrations of angelicin (0 μM to 1 mM) followed by UV exposure. While a majority of the angelicin-modified plasmid DNA molecules migrated as single strands, we did observe a small fraction of the DNA that migrated much slower than the single-stranded species suggestive of interstrand cross-links in concordance with the observation made by Lown and Sim [28]. We hypothesize that this small fraction of cross-links was sufficient to cause the rapid decay of nanopores. However, despite this reduced throughput, we were able to sequence and align ∼64k reads with an average read coverage of 13 from the positive control sample modified with 500 μM angelicin (Supplementary Fig. S1C and Supplementary Table S1).

### Identification of angelicin modification from the nanopore current signal

Using the aligned current signal data from the 500 μM positive and negative control samples, we compared the distribution of current signal values for all combinations of 6-base-long *k*-mers including those with the intercalation motif for angelicin (5′-TA, 5′-AT, 5′-TG, and 5′-GT). A subset of *k*-mers containing the 5′-TA showed a secondary peak in the positive control due to changes in current signals (Fig. 1B and C). In contrast, *k*-mers without the intercalation motif showed no detectable changes in current signals between the positive and negative controls (Fig. 1D and Supplementary Fig. S1D and E). We were somewhat surprised that we only observed a shift in 5′-TA *k*-mers and no discernible shifts in current with 5′-AT, 5′-TG, or 5′-GT containing *k*-mers that should also be potentially modifiable. However, psoralens have also been shown to preferentially cross-link 5′-TA dinucleotides significantly over the rest [25, 26]. It is possible that non-TA *k*-mers were not being modified in our experimental settings or the current shifts were too subtle to be detected. We also observed that the signal shifts in current were more prominent in *k*-mers where the TA dinucleotide was located at the 5′ end of the *k*-mer sequence and consequently were more easily detected by our statistical methodology.

However, since we did observe a current signal shift in *k*-mers with the 5′-TA dinucleotide and given previous work showing that other base modifications, e.g. methylation, alter the current signal [14, 17], we concluded that this secondary distribution was due to angelicin-modified DNA. We also observed a subset of *k*-mers with multiple 5′-TA motifs that showed three total peaks, suggesting both single and double modifications by angelicin on these *k*-mers (Fig. 1B). However, a majority of TA-containing *k*-mers did not show any shifts in the signal distribution between the negative and positive controls (Supplementary Fig. S1F).

After generating a sequence logo for *k*-mers with multiple signal peaks, we found the 5′-TA motif as expected, but more surprisingly we found that the detection was limited primarily to the more specific motif 5′-TAT, which may explain why other TA-containing *k*-mers did not show multiple signal peaks after intercalation of angelicin (Fig. 1E). From the 20, 100, and 500 μM titrations of angelicin treatment on purified genomic DNA, we found that the number of informative *k*-mers directly correlated with the amount of angelicin the DNA was treated with (Supplementary Fig. S1G). However, at higher angelicin concentrations, we also had a significantly faster loss of nanopores (Supplementary Fig. S2D). Therefore, we did not treat or sequence DNA with angelicin concentrations >500 μM.

### Identification of angelicin modification using a neural network model

Due to only being able to detect distinct angelicin-modified signal distributions for 14% of TA-containing *k*-mers and 4% of all *k*-mers based on signal density (Supplementary Fig. S1F), we hypothesized that angelicin modification could more easily be detected using a machine learning model to observe changes in nanopore current signal across a larger window of bases. To map single-molecule chromatin accessibility at nucleosome resolution, we trained and tested a neural network model we call NEMO, to predict angelicin modifications from signal picoamp data without the information of underlying bases. It then maps the modification scores from signal space to sequence space based on the Dorado move-out table.

**Figure 2.**
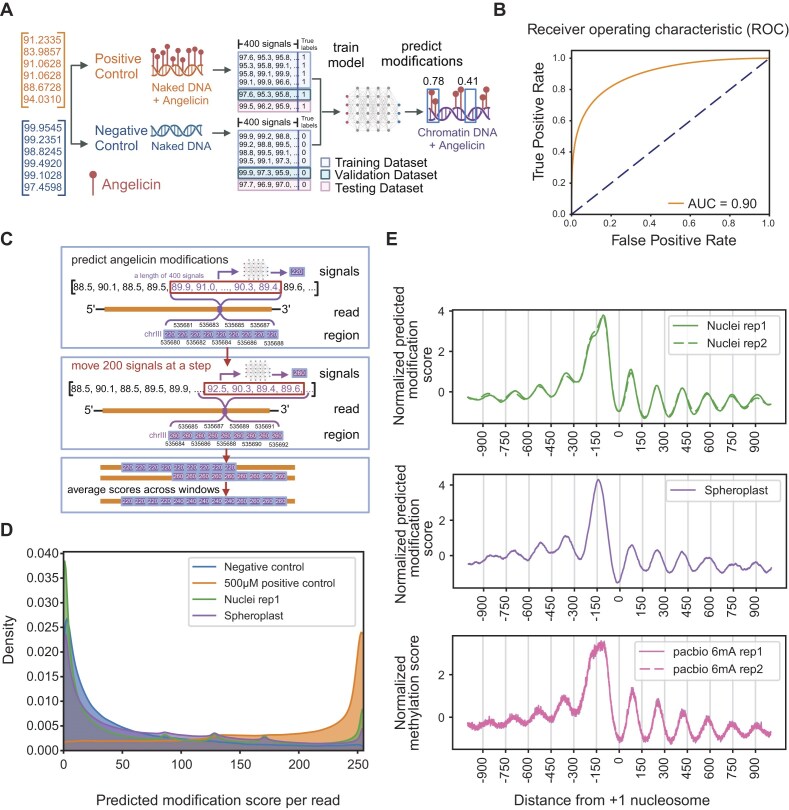
Angelicin modification scoring from the neural network model identifies expected patterns of nucleosome occupancy near TSSs. (**A**) A schematic of the neural network model trained on the nanopore signal currents produced from positive and negative control DNA sequencing. Created in BioRender. Bai, G. (2025) https://BioRender.com/vi3b7nd (**B**) ROC curve showing the performance of the trained model on the stand-alone test dataset. (**C**) A schematic showing how modification probability is predicted from a window of 400 signals per read. The scores were mapped to the genome reference and averaged per position for each read. Created in BioRender. Bai, G. (2025) https://BioRender.com/h71e335 (**D**) Density of average predicted scores per read for negative control DNA, positive control DNA, nuclei chromatin replicate 1, and spheroplast chromatin data. (**E**) Aggregate modification probability predicted by NEMO on angelicin-modified chromatin (top and middle) and PacBio 6mA methylated chromatin (bottom; data from [43]) for 2000 base pairs centered on +1 nucleosome dyad.

In the hyper parameter tuning step, we tested three different model architectures with input signal lengths of 400 or 100. Since we were interested in identifying the presence or absence of nucleosomes, we picked a 400-signal measurement window as the maximum input window, which corresponds to ∼75 bp, or half of a nucleosome footprint (Supplementary Fig. S3A). The one-dimensional residual neural network (ResNet1D) [39] with an input signal length of 400 outperformed the others based on validation accuracy (Supplementary Fig. S3B and C). ResNet1D has been used to monitor electrocardiogram signal data in intensive care units [39]. Considering the analogous nature of electrical current measured by electrocardiograms and ONT flow cells, ResNet1D is ideal for learning signal changes caused by nucleic acid modifications. To infer angelicin-modified regions, we trained the ResNet1D model directly from windows of consecutive nanopore signals (Fig. 2A). Our positive and negative control data were used to train, validate, and test the classification ability of the neural network model. Our model was able to distinguish signal currents from positive and negative control data with an area under the receiver operating curve (AUC) of 0.9 in the stand-alone test dataset (Fig. 2B). This represents a relatively high TPF and low FPR for detecting angelicin modification sites (Supplementary Fig. S3D).

### Identification of angelicin-modified DNA from nuclei and intact cells using NEMO

To evaluate the use of angelicin as a probe for chromatin structure, we treated either yeast nuclei or yeast spheroplasts with 500 μM angelicin followed by UVA irradiation (Fig. 1A). Following angelicin treatment of yeast chromatin in nuclei or spheroplasts, high molecular weight DNA with a mean length of ∼40 kb was extracted (Supplementary Fig. S1B). DNA libraries were constructed, sequenced, and basecalled the same way as the control samples. Overall, the median read length was ∼3 kb, with a median of 100k aligned reads (primary alignments) and a median base phred quality score of 22 (range from 0 to 40) (Supplementary Fig. S1C and Supplementary Table S1).

We next applied NEMO to predict accessible regions in angelicin-modified chromatin data. Individual reads were scanned using a 400-signal sliding window with a step size of 200 signals. Prediction scores were assigned to every base represented by the signal window, and then scores across multiple windows for each base were averaged (Fig. 2C). This corresponds to an average read-level resolution of ∼20 bp. After applying the model to chromatin from both nuclei and spheroplasts, we saw that both showed a pattern of predicted accessibility that fell between the positive and negative control datasets, indicating an intermediate pattern of modification as expected with some genomic regions blocked from intercalation by nucleosomes (Fig. 2D). The fact that the spheroplast data showed any predicted modification indicates that angelicin is, in fact, cell-permeable and could successfully modify chromatin *in vivo*.

### Identification of chromatin structure using angelicin modification

The region around the TSS of a transcriptionally active gene shows a characteristic pattern of chromatin accessibility upstream of the TSS. The DNA is rendered generally accessible, allowing general transcription factors and RNA polymerase II to bind. Downstream of the TSS is the +1 nucleosome, followed by a regular pattern of positioned nucleosomes interspersed with accessible linker regions. Furthermore, the first nucleosome is expected to be the most well positioned, with subsequent downstream nucleosomes being less well positioned [49]. After generating accessibility predictions for each read from the neural network, we averaged all predictions for a window of ±1000 bp around each +1 nucleosome at protein-coding genes and normalized its mean and standard deviation (Fig. 2E). From this metagene plot, we found that both the angelicin-modified nuclei and spheroplast chromatin samples closely replicated the pattern of accessibility expected from previous short-read methods and with a recently published orthologous long-read method using EcoGII to detect chromatin accessibility with PacBio sequencing [43] (Fig. 2E). We also found that this method gave us similar aggregate modification patterns between two of the three nuclei replicates (Fig. 2E). Replicate 3, due to a poor Pearson correlation with the expected nucleosome periodicity and a failure to show the expected aggregate nucleosome patterns, failed our QC (Supplementary Table S2). However, we did not detect any obvious differences between this replicate and the other two replicates when we compared the read quality and coverage between them and are thus unable to ascribe a reason for why replicate 3 failed.

While investigating the cause of a small dip at −150 bp in the metagene plot from nuclei, we found that yeast TSSs are mostly enriched for the 5′-TA motif except at position −150 bp upstream of the +1 nucleosome, which would bias the angelicin modification efficiency (Supplementary Fig. S3E). Curiously, we did not observe this dip in our spheroplast sample and future investigations will explore whether this is a technical bias caused by lower read coverage of spheroplast data or a biological difference, given that we are modifying chromatin, *in vivo*, with spheroplasts.

### Identification of heterogeneous chromatin accessibility patterns at individual genes

**Figure 3.**
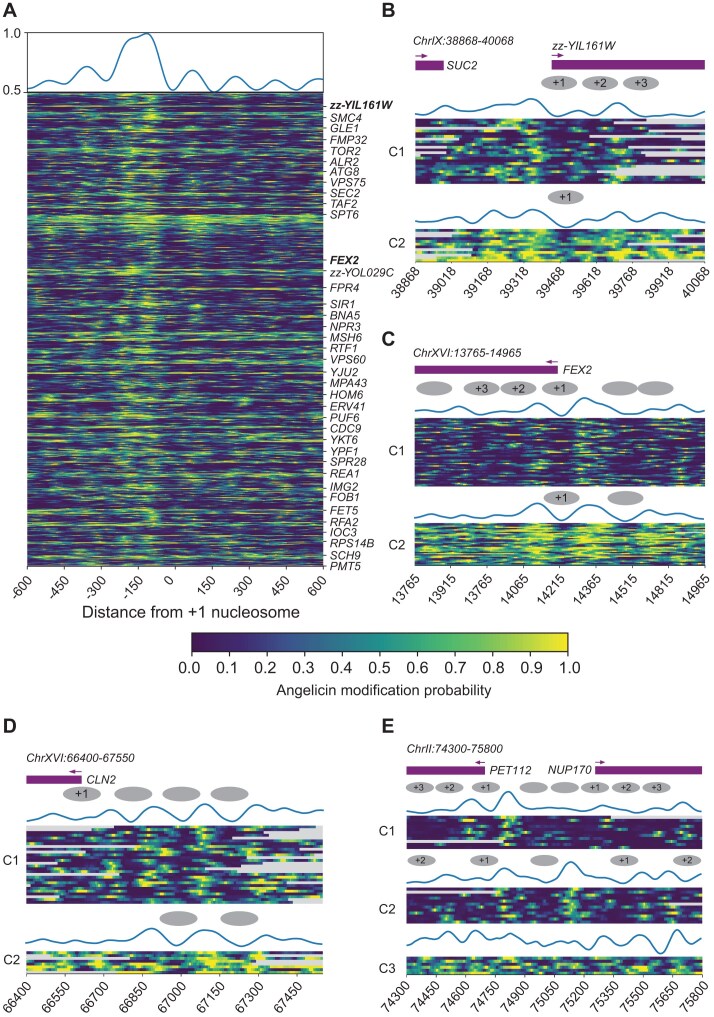
Single-molecule analysis of chromatin structure using SMAdd-seq. Each row is a single DNA molecule covering the locus. The heatmap shows the probability of angelicin modification where yellow is likely modified and blue is unlikely unmodified. The reads are grouped using *k*-means clustering of the modification scores. The top wiggle shows average modification scores per cluster after minimum–maximum normalization. Ovals in the schematic represent the positioning of the nucleosomes. (**A**) Top genes with well-positioned +1 nucleosomes at TSSs. Top 38 genes were shared between yeast nuclei replicate 1 and replicate 2 samples replicate 1 data were used for visualization. Genes were ranked by the Pearson correlation of its modification scores at TSSs with whole genome aggregated modification scores at TSSs. Showing replicate 1 (number of reads = 925). (**B**) zz-YIL161W (ChrIX:38868–40068, +) gene promoter region centered at +1 nucleosome dyad. Reads were grouped into two clusters. Showing replicate 2 (number of reads = 33). (**C**) FEX2 (ChrXVI:13765–14965, −) gene promoter region centered at +1 nucleosome dyad. Reads were grouped into two clusters. Showing replicate 1 (number of reads = 86). (**D**) The CLN2 promoter (ChrXVI:66400–67550, −). Reads were grouped into two clusters. Showing replicate 2 (number of reads = 34). (**E**) The NUP170 TSS (ChrII:74300–75800, +). Reads were grouped into three clusters. Showing replicate 1 (number of reads = 29).

To examine patterns of accessibility on a single-gene, single-molecule level, we examined our two nuclei replicates and spheroplast data, which showed expected nucleosome profiles in aggregate. We identified high-coverage genes with well-positioned +1 nucleosomes by calculating the Pearson correlation of the accessibility pattern at the gene level with the accessibility pattern at the metagene level (Supplementary Fig. S4 and Supplementary Table S2). Although the Pearson correlation of accessibility patterns between the two nuclei replicates, as well as between nuclei and spheroplast sample, was significant (Supplementary Fig. S5G and H), the correlation in read coverage remained low (Supplementary Fig. S5I and J). We attribute this to our low sequencing throughput. We identified 38 genes that had a high read coverage and also showed a significant correlation of their accessibility pattern in both nuclei replicates (replicates 1 and 2) and visualized the ±600-bp window around the +1 nucleosome for every read aligned to these genes (Fig. 3A). We also identified 57 genes with high-coverage and a high Pearson correlation in spheroplast data (Supplementary Fig. S6B). However, due to the significant variability of high coverage genes between samples, there was no overlap between the 38 genes identified in nuclei and the 57 genes from spheroplasts. We show that the angelicin modification-based accessibility predictions represent the expected chromatin structure even at the single-read level. The promoter and +1 and +2 nucleosomes can clearly be seen from the read accessibility across these top genes.

When we zoom into specific gene loci, we can cluster the reads aligned to specific loci based on the accessibility predictions. *zz-YIL161W* and *FEX2* are two genes with well-structured chromatin based on our analysis (Fig. 3B and C, and Supplementary Fig. S6C and D). Both genes have two clusters of reads with distinct patterns of accessibility that are reminiscent of the structural heterogeneity previously observed for the *PHO5* promoter of yeast by psoralen-EM analysis [3]. In *zz-YIL161W*, we observe the +1, +2, and +3 nucleosomes in C1, while only the +1 nucleosome is observed in C2, indicating more transcriptionally active chromatin (Fig. 3B). In the *FEX2* gene, we observe well-positioned nucleosome arrays in C1 indicating a transcriptionally repressive state, while in C2 we only observe the +1 nucleosome and an upstream nucleosome representing a transcriptionally active state of this gene. C2 is also more accessible overall than C1 (Fig. 3C).

We can also identify changes in chromatin accessibility in regions other than the TSS. The *CLN2* promoter is a well-studied cell cycle-regulated promoter that has a large nucleosome-depleted region (NDR) upstream of the TATA box [50]. After *k*-means clustering of the predicted modification scores at this locus, we observe a cluster (C1) that shows three well-positioned nucleosomes in the promoter region and little accessibility downstream (Fig. 3D and Supplementary Fig. S6E). The second cluster shows only one nucleosome positioned in the promoter and greater accessibility in the gene body, indicating a more transcriptionally active subset of cells regulated by transcription factors binding at the upstream NDR.

We can also identify clustering patterns that are not dependent on overall accessibility. *PET112* and *NUP170* are divergent neighboring genes (Fig. 3E and Supplementary Fig. S6F). After clustering the reads at this locus, we identify three distinct clusters—one with only the *PET112* promoter open (C1), one with both promoters open (C2), and one with higher accessibility across the locus (C3). Compared to C1, both genes in C2 were more accessible, possibly due to greater transcriptional activity. These genes represent examples of a range of chromatin accessibility patterns that can be investigated using SMAdd-seq.

## Discussion

As previously described [25], we also find that angelicin can modify thymine bases in a 5′-TA context on single strands of DNA and our work has shown that these strands can be sequenced on nanopores. We also show that angelicin-modified *k*-mers had a distinct current signal compared to unmodified *k*-mers. Additionally, we trained a neural network model for estimating the probability of DNA modification by angelicin from nanopore signal measurements. Furthermore, our computational method may also be useful for detecting any kind of modification with a matched positive and negative training dataset, beyond its application in angelicin modification detection. Although angelicin modification caused distinct signal shifts in a small fraction of *k*-mers, our machine learning model was, on average, able to detect open and closed chromatin, different patterns of chromatin accessibility, and patterns of intramolecular correlation. These methods allow us to detect the chromatin accessibility on both a genome-wide level and the single-molecule level at specific loci. We also show that angelicin can modify DNA and accurately mark chromatin accessibility *in vivo*, without the need for nuclei extraction.

The biggest challenge we have faced with nanopore sequencing of angelicin-modified DNA is the sparseness of data, primarily due to a combination of incomplete modification of accessible modifiable sites and blockage of the pores, presumably due to DNA cross-linking. We were also surprised that we did not detect 5′-AT, 5′-TG, or 5′-GT dinucleotide adducts in our sequencing data. However, both, Esposito *et al.* [26] and Komura *et al.* [25] also observed a similar dinucleotide bias in their data with psoralen suggesting that the overwhelming preference for modifying 5′-TA that we observed may be a more common property of furocoumarins. Some of the incomplete angelicin modification may also be due to modification of only one strand and not the other in a TA/AT context, because angelicin can only covalently bond with a single thymine base in one strand of the DNA. As a result, we failed to sequence the strand containing the modification half the time with standard nanopore sequencing. This means that even in our positive control, we are not detecting modifications for all modifiable sites. This is a nontrivial problem, especially for the neural network-based model, as machine learning models depend highly on good-quality training data. Other groups have used synthetic DNA with modified bases at known sites to train similar models. However, we could not find any available protocols or companies that could generate synthetic angelicin-modified DNA templates. Future work may utilize the newly developed nanopore duplex sequencing to sequence both strands of DNA [51, 52], increasing the probability of sequencing the modified *k*-mer at each modifiable position. However, at the moment, the duplex sequencing method is currently not sufficiently high-throughput enough to generate datasets for network training [51, 52].

Previous work has suggested that the chemistry of angelicin should not allow for the formation of cross-links [25, 27], unlike the angelicin analog, psoralen, which mostly forms interstrand cross-links [3]. However, we observed a small fraction of angelicin-treated DNA containing interstrand cross-links. This result combined with the more rapid decay of flow cell pores on samples with angelicin treatment leads us to hypothesize that the interstrand cross-links in the DNA cannot pass through the pores, thus clogging them and reducing the throughput of the flow cell. One way to alleviate this issue would be to incubate DNA at elevated temperature and basic pH to break interstrand cross-links. Base treatment has been successfully used before to break DNA cross-links formed by psoralen [53]. However, the adapter protein required to ratchet the DNA through the nanopore during sequencing will not withstand such harsh treatment, thus precluding this option. Conversely, treating the modified DNA with alkali prior to adapter ligation to reverse cross-links would render it single-stranded, and since the adapter ligates only to dsDNA, perfectly renaturing long stretches of high-complexity DNA would be challenging.

Other options include modifying angelicin itself as well as generating alternative angular structures of other furocoumarin derivatives to determine how they traverse the nanopore and utilizing this information to synthesize and test alternative small molecules that can be used as probes for visualizing the chromatin landscape [54, 55]. Other less damaging furocoumarin derivatives will also enable this method to be extended to alternative long-read sequencing methods like PacBio sequencing since DNA polymerases are currently unable to polymerize through an angelicin-modified template (data not shown).

Despite these challenges, our current protocol allowed us to detect chromatin accessibility both at the genome-wide level and at the single locus level. There are other additional benefits of using this small molecule as opposed to enzymes in probing chromatin structure. Compared to enzyme-based approaches, angelicin modification is significantly cheaper per Gb of sequence generated: approximately $1 for angelicin compared to $100 for the commercial EcoGII methyltransferase based on throughput from [43]. Furthermore, angelicin is naturally found only in plant cells and therefore acts as a fully exogenous DNA modification in fungi and animal cells [56]. Other approaches use GpC methyltransferases to label genomes that also have endogenous CpG modification, which results in the exclusion of methylation data in a GCG context due to ambiguity between native methylation and exogenous modification [14]. While we have not yet tested the permeability of angelicin through the yeast cell wall, there is a report in the literature where the authors used intact yeast cells to study excision repair in cells exposed to angelicin and psoralen [27]. We plan on testing whether angelicin can be directly added to intact yeast cells in future experiments. The membrane permeability of furocoumarins, however, has been well demonstrated [24], and this feature allows one to probe chromatin accessibility in a variety of cell types without isolating nuclei, which has been previously shown to affect chromatin structure accessibility [21, 22]. Removing the step of nuclei isolation can make accessibility probing more amenable to low-input tissue samples or other single-cell analyses. We do acknowledge that the irradiation step on ice may induce a cold stress on cells, but we argue that this treatment is insignificant compared to the perturbations that occur during cell lysis, pipetting, centrifugation, and cold incubations during nuclei purification and storage. While the angelicin modification protocol is in need of further optimization, we show that nanopore sequencing of angelicin-modified chromatin is a highly feasible method for probing chromatin structure *in vivo* and may facilitate chromatin studies in cells or tissue that have been difficult to probe.

## Supplementary Material

gkaf671_Supplemental_Files

## Data Availability

Raw nanopore signal data are deposited at https://zenodo.org/records/15122707. Basecalled nanopore sequencing data and alignment files are available under BioProject PRJNA1084879. Data and codes for regenerating figures are available at https://github.com/baigal628/smaddseq_manuscript. Our computational model NEMO is available at https://github.com/baigal628/NEMO and Zenodo https://zenodo.org/records/15692384.
