# Supplementary material for: SMAdd-seq: probing chromatin accessibility with small molecule DNA intercalation and nanopore sequencing
Source: Nucleic Acids Res. 2025 Jul 19;53(14):gkaf671. doi: 10.1093/nar/gkaf671 (PMC12276009; doi:10.1093/nar/gkaf671)
Supplement: gkaf671_Supplemental_Files [file gkaf671_supplemental_files.zip › Supplementary Material_250613.pdf]

SUPPLEMENTARY FIGURES

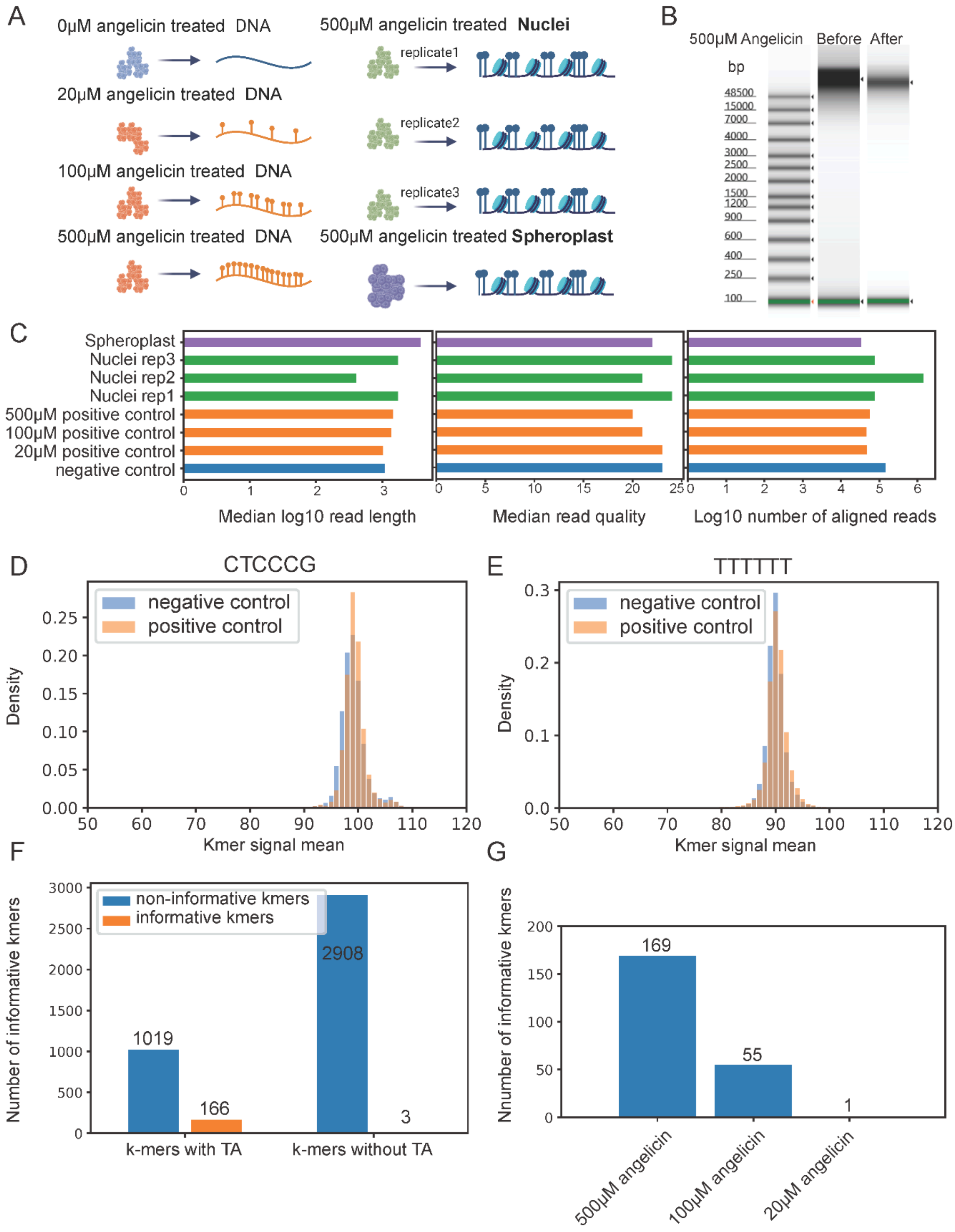

Supplementary Figure 1. Data quality control and k-mer analysis. (A) A summary of yeast sequencing data generated for the SMAdd-seq experiments, which includes a titration experiment on genomic DNA with angelicin concentration from 0μM, 20μM, 100μM to 500μM (left), triplicates of yeast nuclei treated with 500μM angelicin and one replicate of spheroplast treated with 500μM angelicin. (B) A TapeStation profile of spheroplast DNA treated with 500μM angelicin before (left lane) and after (right lane) library preparation. This sample was used to prepare ONT libraries with the SQK-LSK110 sequencing kit. (C) Bar plots showing the quality control metrics from nanopore sequencing data. Chromatin rep3 is dropped from downstream analysis due to the lack of genes with well-positioned nucleosomes. (D & E) Histograms of the nanopore signal currents produced from T containing unmodifiable k-mers in yeast DNA that had been either treated with UV light only (blue: negative control) or with angelicin and UV for (orange: positive control). No shift in signal currents were observed from positive control data. (F) Bar plots showing number of informative k-mers (orange: has at least one shifted peak in positive control data) and non-informative k-mers (blue: has no shifted peak in positive control data) grouped by whether the kmer contains a modifiable TA dimer. (G) Bar plots showing increased number of k-mers with detectable modification signal with increased angelicin concentration.

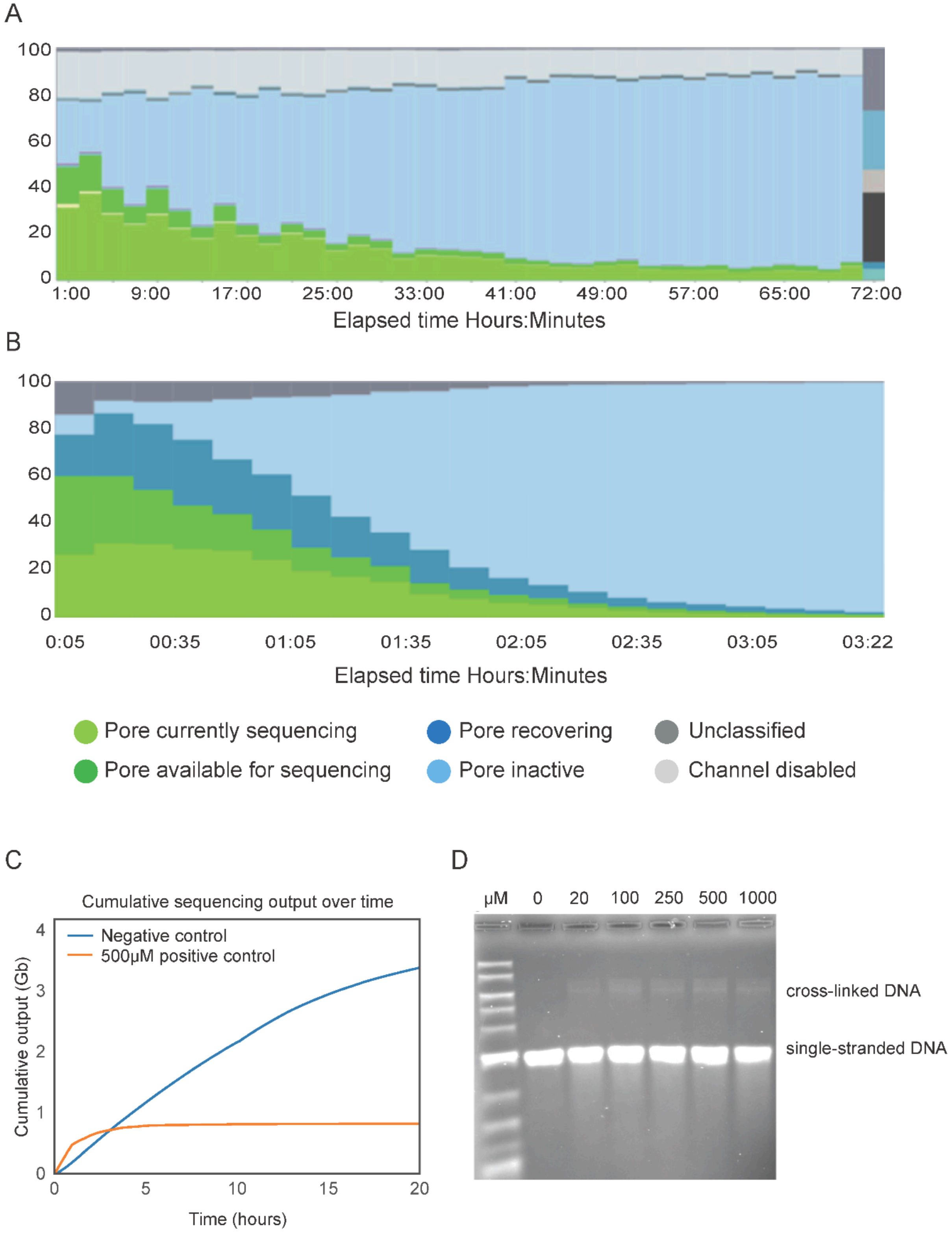

Supplementary Figure 2. DNA crosslinking due to angelicin modification leads to reduced throughput of flow cells. (A & B) The histograms show percent activity of nanopores

during a sequencing run with (A) Unmodified DNA, x-axis has a maximum value of 72 hours and (B) DNA modified with 500μM angelicin, x-axis has a maximum value of ~3 hours. (C) Cumulative sequencing output for a sequencing run of DNA treated with either 0μM or 500μM angelicin. X-axis indicates the time elapsed for sequencing. (D) Denaturing alkaline agarose gel electrophoresis of linearized plasmid BlueScript (pBS) modified with varying concentrations of angelicin (0μM to 1000μM). A majority of the DNA migrated as single stranded DNA (lower band); however, a small amount of double-stranded DNA (upper band) was observed in lanes with angelicin-modified DNA.

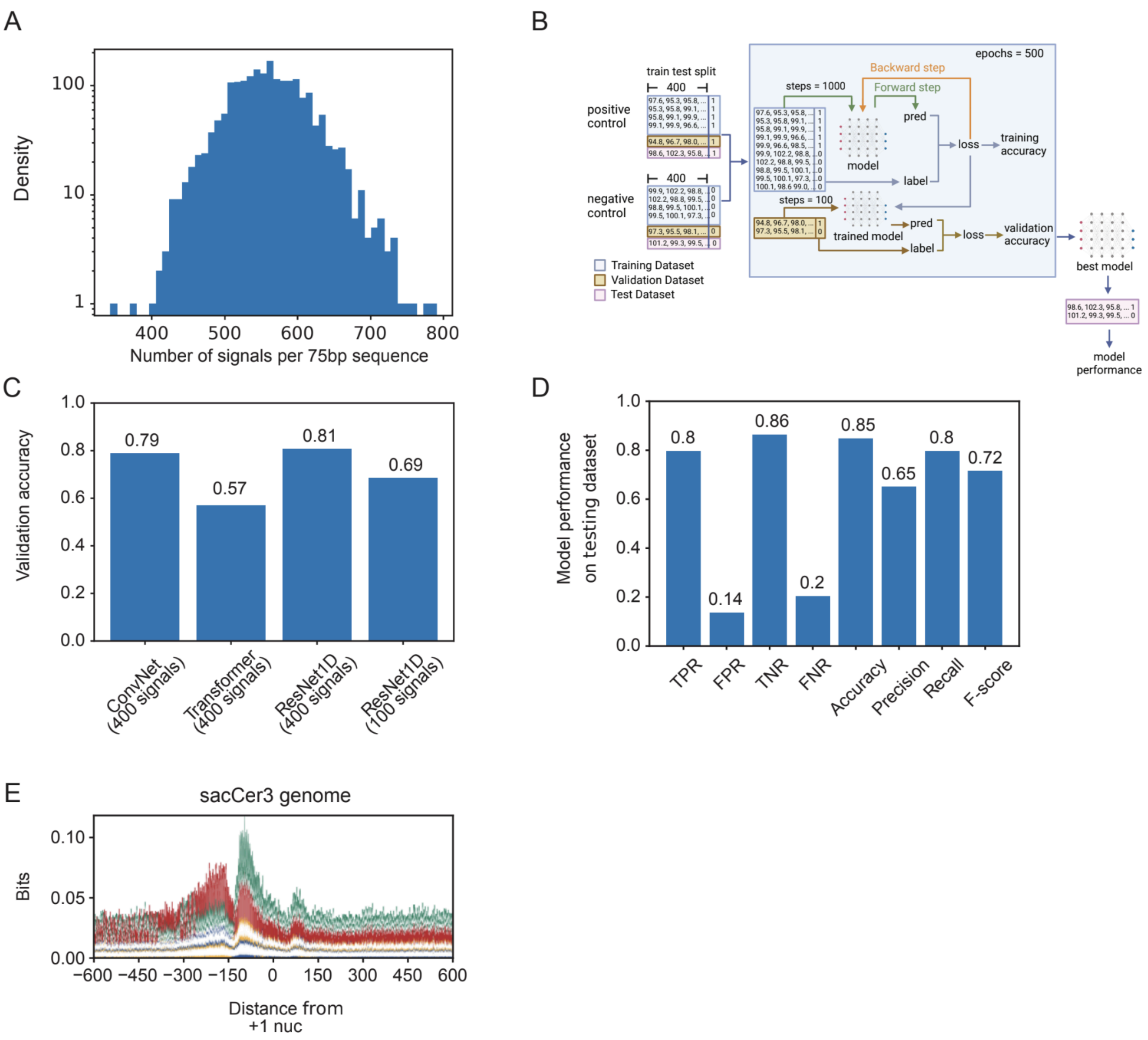

Supplementary Figure 3. Detailed illustration of deep learning model training and model performance evaluation. (A) Number of signal scores per 75bp window after eliminating 6-mers with > 10 scores assigned to them (indicates DNA pausing, causes outliers). (B) A schematic of the model training process. Signal currents from each control sample were split into training (60%), validation (20%), and testing (20%) datasets. After training 500 epochs, the best model was chosen based on the best prediction accuracy on validation dataset. The final model performance is evaluated on the testing dataset. (C) Bar plot comparing prediction accuracy on validation dataset when using different neural network architectures and different data input sizes. (D) Bar plot showing final model performance on testing dataset. TPR=true positive rate, FPR=false positive rate, TNR=true negative rate, FNR=false negative rate (E) Sequence logo showing motif enrichment at transcription start sites in the yeast genome. A depletion of TA was observed at 150 bp upstream +1 nucleosome dyad (A: Green, T: Red, C:Blue, G: Yellow)

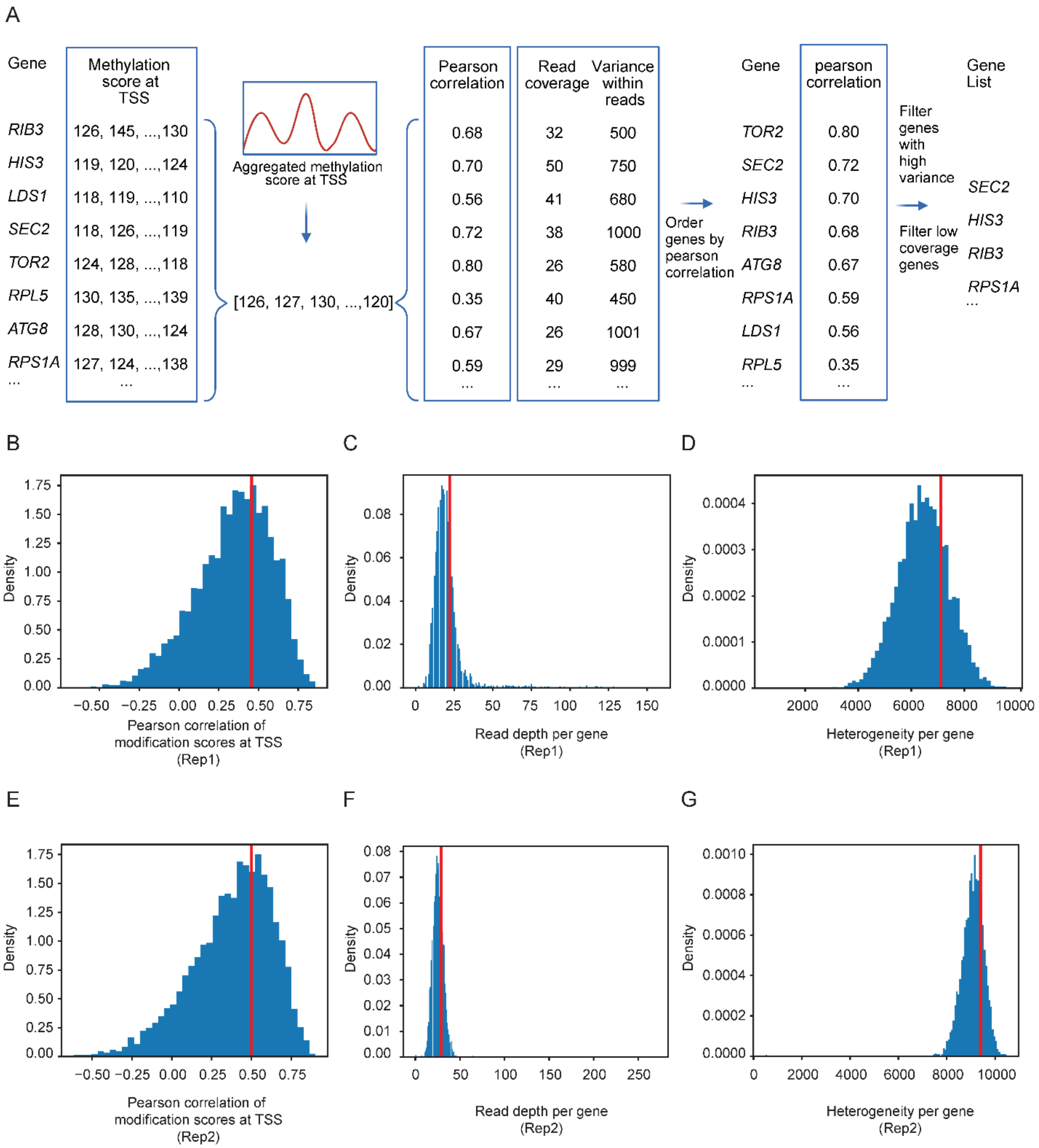

Supplementary Figure 4. Selection of genes with well-positioned nucleosomes at transcription start sites. (A) A schematic illustrating genes with well-positioned nucleosomes were selected based on the Pearson correlations between their angelicin modification scores at TSS and the genome-wide aggregated angelicin modification scores. (B-G) Histogram showing the distribution of Pearson correlation of modification scores at the promoter of a single gene with the aggregate promoter modification pattern, read depth per gene and heterogeneity between reads per gene across all annotated genes in the Nuclei rep1 sample (B, C & D) and Nuclei rep1 sample (E, F & G). Red lines indicate the 75 percentile cutoffs used for filtering genes.

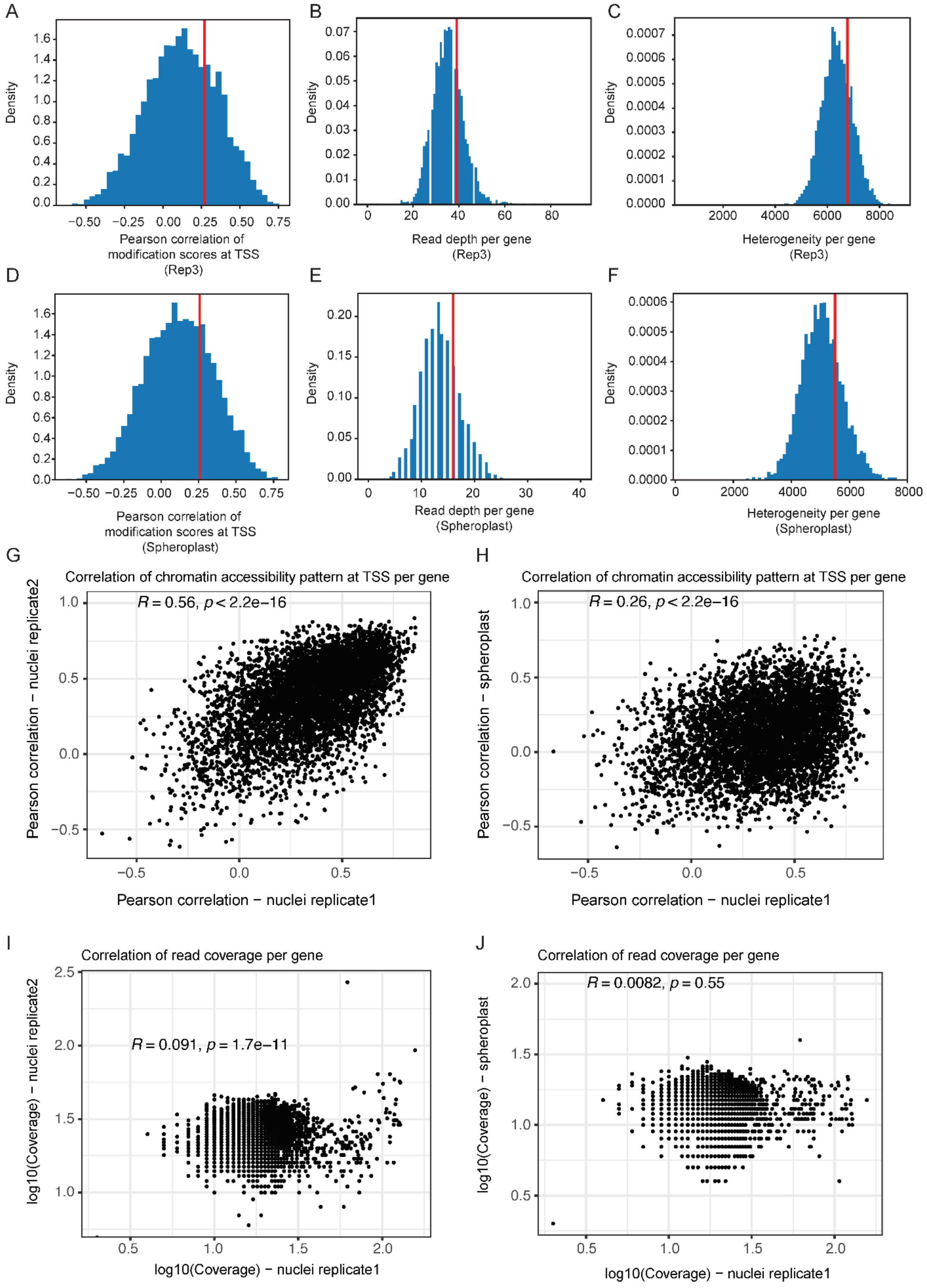
A
Density
Pearson correlation of
modification scores at TSS
(Rep3)
B
Density
Read depth per gene
(Rep3)
C
Density
Heterogeneity per gene
(Rep3)
D
Density
Pearson correlation of
modification scores at TSS
(Spheroplast)
E
Density
Read depth per gene
(Spheroplast)
F
Density
Heterogeneity per gene
(Spheroplast)
G
Correlation of chromatin accessibility pattern at TSS per gene
R = 0.56, p < 2.2e−16
Pearson correlation − nuclei replicate2
Pearson correlation − nuclei replicate1
H
Correlation of chromatin accessibility pattern at TSS per gene
R = 0.26, p < 2.2e−16
Pearson correlation − spheroplast
Pearson correlation − nuclei replicate1
I
Correlation of read coverage per gene
R = 0.091, p = 1.7e−11
log10(Coverage) − nuclei replicate2
log10(Coverage) − nuclei replicate1
J
Correlation of read coverage per gene
R = 0.0082, p = 0.55
log10(Coverage) − spheroplast
log10(Coverage) − nuclei replicate1

Supplementary Figure 5. Quality control of nucleosome configurations in chromatin datasets

(A-F) Histogram showing the distribution of Pearson correlation of modification scores at the promoter of a single gene with the aggregate promoter modification pattern, read depth per gene and heterogeneity between reads per gene across all genes with annotated +1 nucleosomes in the nuclei replicate 3 sample (A, B & C) and spheroplast sample (D, E & F). Red lines indicate the 75 percentile cutoffs used for filtering genes. (G) Pearson correlation of chromatin accessibility pattern at TSS (calculated as Pearson correlation between per gene angelicin modification and genome-wide accessibility pattern) between nuclei replicate 1 and replicate 2 data. (H) Pearson correlation of chromatin accessibility pattern at TSS (calculated as Pearson correlation between per gene angelicin modification and genome-wide accessibility pattern) between nuclei replicate 1 and spheroplast data. (I) Pearson correlation of read coverage per gene between nuclei replicate 1 and nuclei replicate 2 data. (J) Pearson correlation of read coverage per gene between nuclei replicate 1 and spheroplast data.

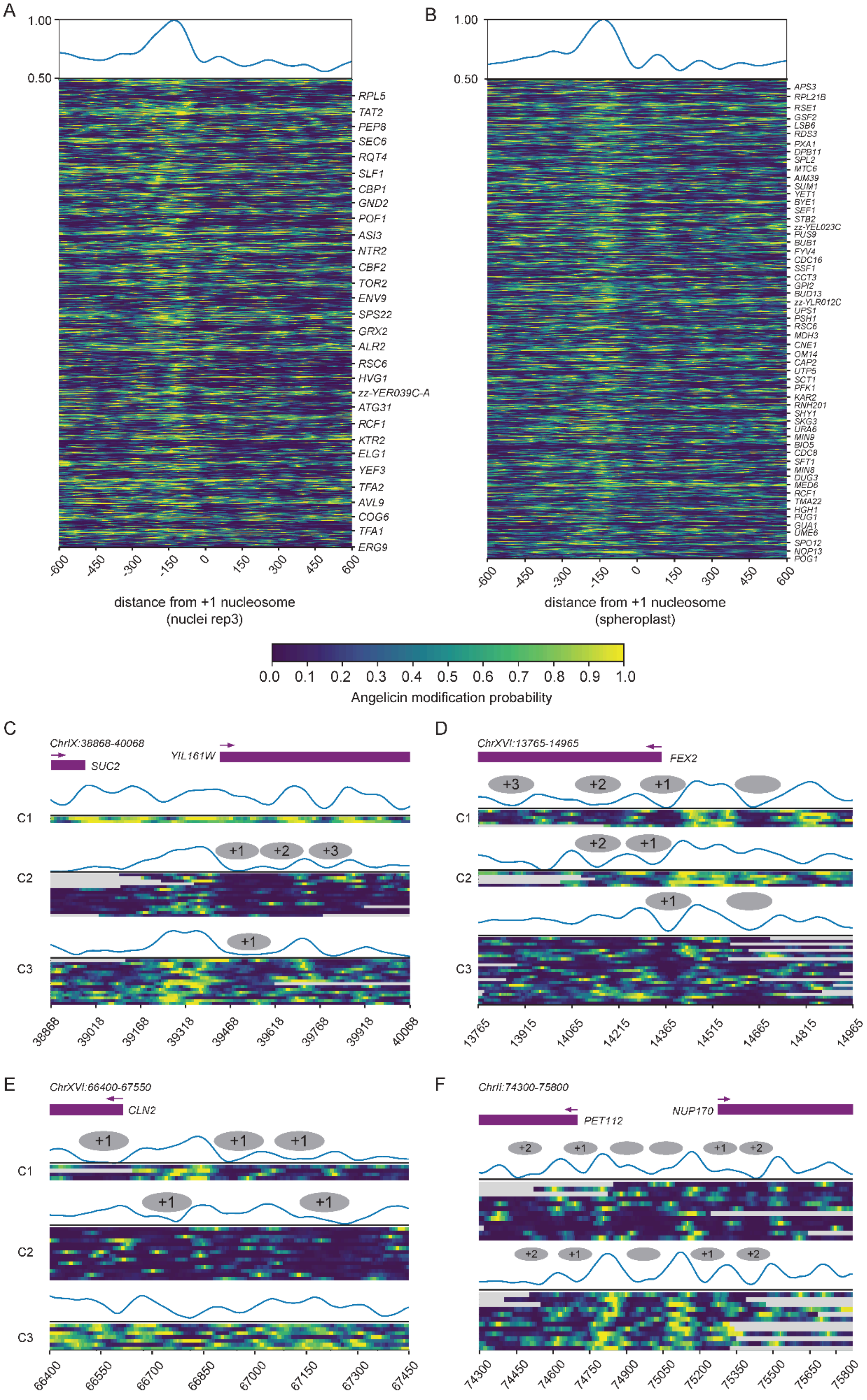
A
1.00
0.50
RPL5
TAT2
PEP8
SEC6
RQT4
SLF1
CBP1
GND2
POF1
ASI3
NTR2
CBF2
TOR2
ENV9
SPS22
GRX2
ALR2
RSC6
HVG1
zz-YER039C-A
ATG31
RCF1
KTR2
ELG1
YEF3
TFA2
AVL9
COG6
TFA1
ERG9
-600 -450 -300 -150 0 150 300 450 600
distance from +1 nucleosome
(nuclei rep3)
B
1.00
0.50
APS3
RPL21B
RSE1
GSF2
LSB6
RDS3
PXA1
DPB11
SPL2
MTC6
AIM39
SUM1
YET1
BYE1
SEF1
STB2
zz-YEL023C
PUS9
BUB1
FYV4
CDC16
SSF1
CCT3
GPI2
BUD13
zz-YLR012C
UPS1
PSH1
RSC6
MDH3
CNE1
OM14
CAP2
UTP5
SCT1
PFK1
KAR2
RNH201
SHY1
SKG3
URA6
MIN9
BIO5
CDC8
SFT1
MIN8
DUG3
MED6
RCF1
TMA22
HGH1
PUG1
GUA1
UME6
SPO12
NOP13
POG1
-600 -450 -300 -150 0 150 300 450 600
distance from +1 nucleosome
(spheroplast)
0.0 0.1 0.2 0.3 0.4 0.5 0.6 0.7 0.8 0.9 1.0
Angelicin modification probability
C
ChrX:38868-40068
SUC2
YIL161W
C1
C2
+1 +2 +3
C3
+1
38868 39018 39168 39318 39468 39618 39768 39918 40068
D
ChrXVI:13765-14965
FEX2
+3 +2 +1
C1
+2 +1
C2
+1
C3
13765 13915 14065 14215 14365 14515 14665 14815 14965
E
ChrXVI:66400-67550
CLN2
+1 +1 +1
C1
+1 +1
C2
C3
66400 66550 66700 66850 67000 67150 67300 67450
F
ChrII:74300-75800
PET112
NUP170
+2 +1 +1 +2
+2 +1 +1 +2
74300 74450 74600 74750 74900 75050 75200 75350 75500 75650 75800

Supplementary Figure 6. Single-molecule analysis of chromatin structure using SMAdd-seq

Each row is a single DNA molecule covering the locus. The heatmap shows the probability of angelicin modification where yellow is likely modified and blue is unlikely unmodified. The reads are grouped using k-means clustering of the modification scores. The top wiggle shows average modification scores per cluster after min max normalization. Gray ovals in the schematic represent the positioning of the nucleosomes. (A) Angelicin modifications in reads mapped to top 30 genes with well-positioned +1 nucleosomes at TSS in yeast nuclei replicate3 (n reads = 1252). Genes were ranked by the Pearson correlation of its modification scores at TSSs with whole genome aggregated modification scores at TSSs from replicate 1. (B) Angelicin modifications in reads mapped to top 57 genes with well-positioned +1 nucleosomes at TSS in yeast spheroplast (n reads = 975). Genes were ranked by the Pearson correlation of its modification scores at TSSs with whole genome aggregated modification scores. (C) zz-YIL161W (ChrIX:38,868-40,068, +) gene promoter region centered at +1 nucleosome dyad. Reads were grouped into 3 clusters. Showing replicate 1 (number of reads = 33). (D) FEX2 (ChrXVI:13765-14965, -) gene promoter region centered at +1 nucleosome dyad. Reads were grouped into 2 clusters. Showing replicate 2 (number of reads = 34). (E) The CLN2 promoter (ChrXVI:66,400-67,550, -). Reads were grouped into 2 clusters. Showing replicate 1 (number of reads = 25). (F) the NUP170 TSS (ChrII:74,300-75,800, +). Reads were grouped into 3 clusters. Showing replicate 2 (number of reads = 24).

SUPPLEMENTAL DATA

Supplemental Data are available at NAR online.

Supplemental Table 1: Quality Control Metrics for Sample Sequencing

Supplemental Table 2: Nucleosome Occupancy Analysis of Individual Genes
